# Supplementary figures and images for: Single-nucleus and single-cell transcriptomes compared in matched cortical cell types
Source: PLoS One. 2018 Dec 26;13(12):e0209648. doi: 10.1371/journal.pone.0209648 (PMC6306246; doi:10.1371/journal.pone.0209648)

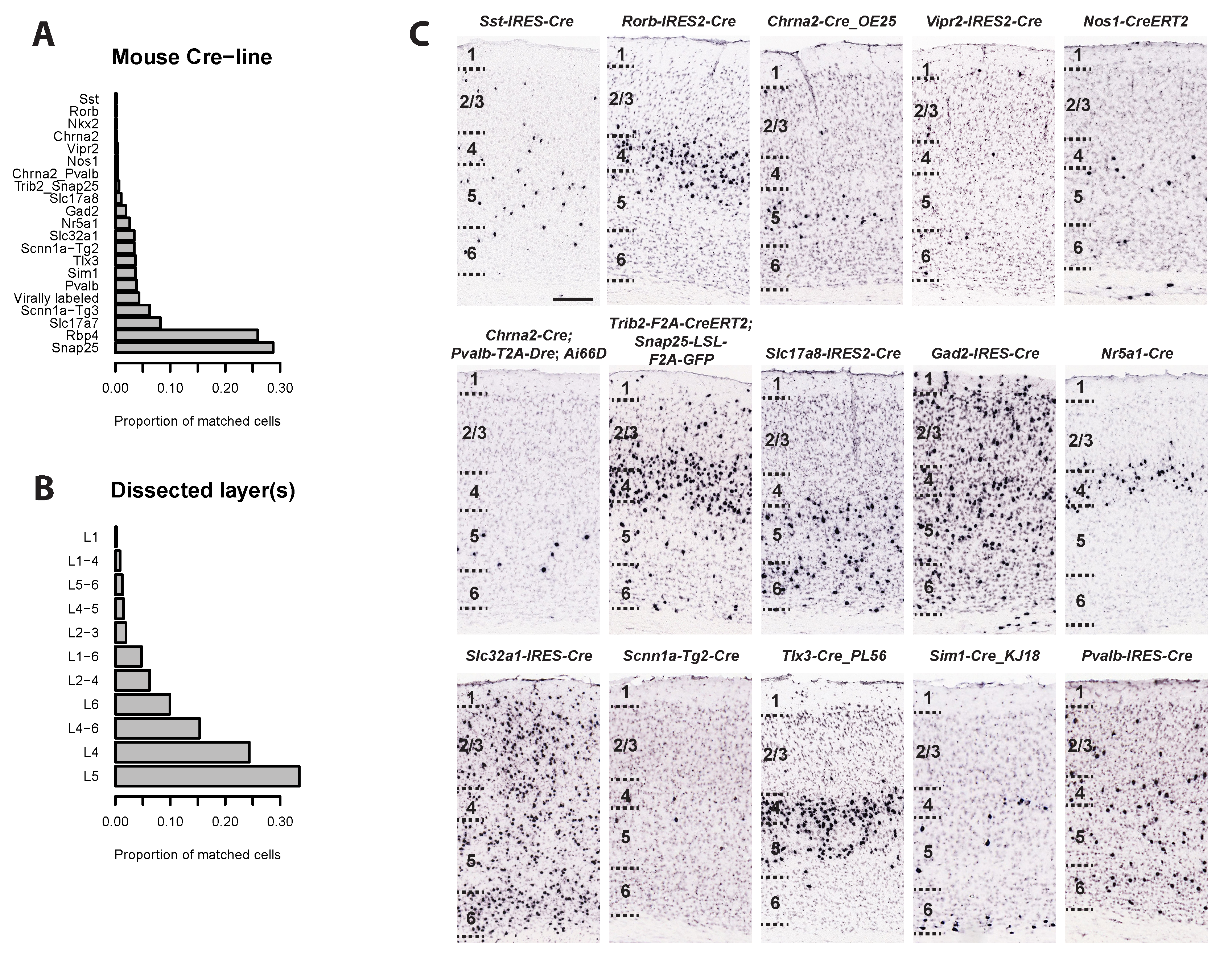

Supplement: S1 Fig — (A) Proportion of matched cells isolated from transgenic mouse lines that label different subsets of cortical neurons. Note that a small number of “virally labeled” cells (<5%) were FAC sorted from wild-type mice based on retrograde labeling by viral injections into various cortical and subcortical structures. (B) Proportion of matched cells dissected from one or more adjacent layers of cortex. (C) ISH images from additional mouse Cre-lines from which the best matching cells were most commonly derived. ISH images show all cortical layers within VISp. All recombinase lines were crossed to either Ai14 or Ai110 [54], except Chrna2-Cre_OE25;Pvalb-T2A-Dre;Ai66D [55], and Trib2-F2A-CreERT2;Snap25-LSL-F2A-GFP [55], for which the reporters are indicated. (TIFF) [file pone.0209648.s001.tiff]

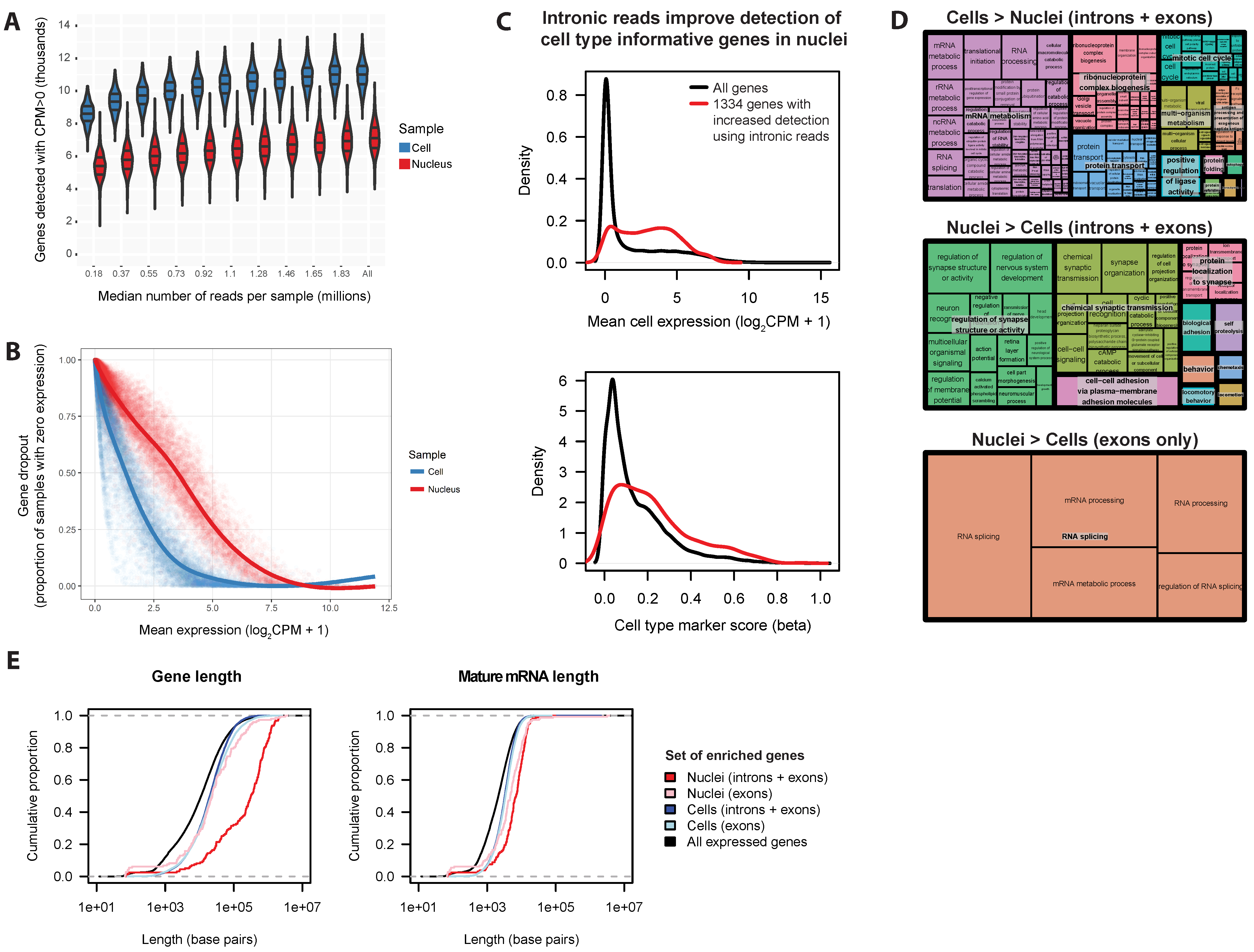

Supplement: S2 Fig — (A) Gene detection violin plots for nuclei and cells at different sub-sampled read depths. Note that while gene detection does not fully saturate, 90% as many genes are detected with 1 million versus approximately 2.5 million (“All”) reads. (B) Rate of gene dropouts in nuclei versus cells (i.e. proportion of nuclei/cells with zero expression) as compared to the average gene expression level across all nuclei and cells. Loess fits to dropout rates of genome-wide genes. (C) Density plots showing the properties of all expressed genes (black lines) and 1334 genes (red lines) that have >25% detection in nuclei using intronic plus exonic reads versus only exonic reads. Mean expression was calculated using only exonic reads in cells, and beta marker scores were calculated for cell clusters as described in the Methods. (D) REVIGO summaries of gene ontology (GO) enrichment of genes enriched in cells or nuclei. Including introns dramatically changes the functional categories of nuclear but not cell enriched genes. (E) Cumulative distribution of genomic and transcript lengths for genes enriched in nuclei and cells (fold change > 1.5) based on expression of exons or introns plus exons. Using introns plus exons, the median genomic length of nuclear enriched genes is 16-fold longer than cell enriched genes. Using exons only, there is no significant difference in genomic lengths (Kolmogorov-Smirnov test P-value = 0.27). (TIFF) [file pone.0209648.s002.tiff]

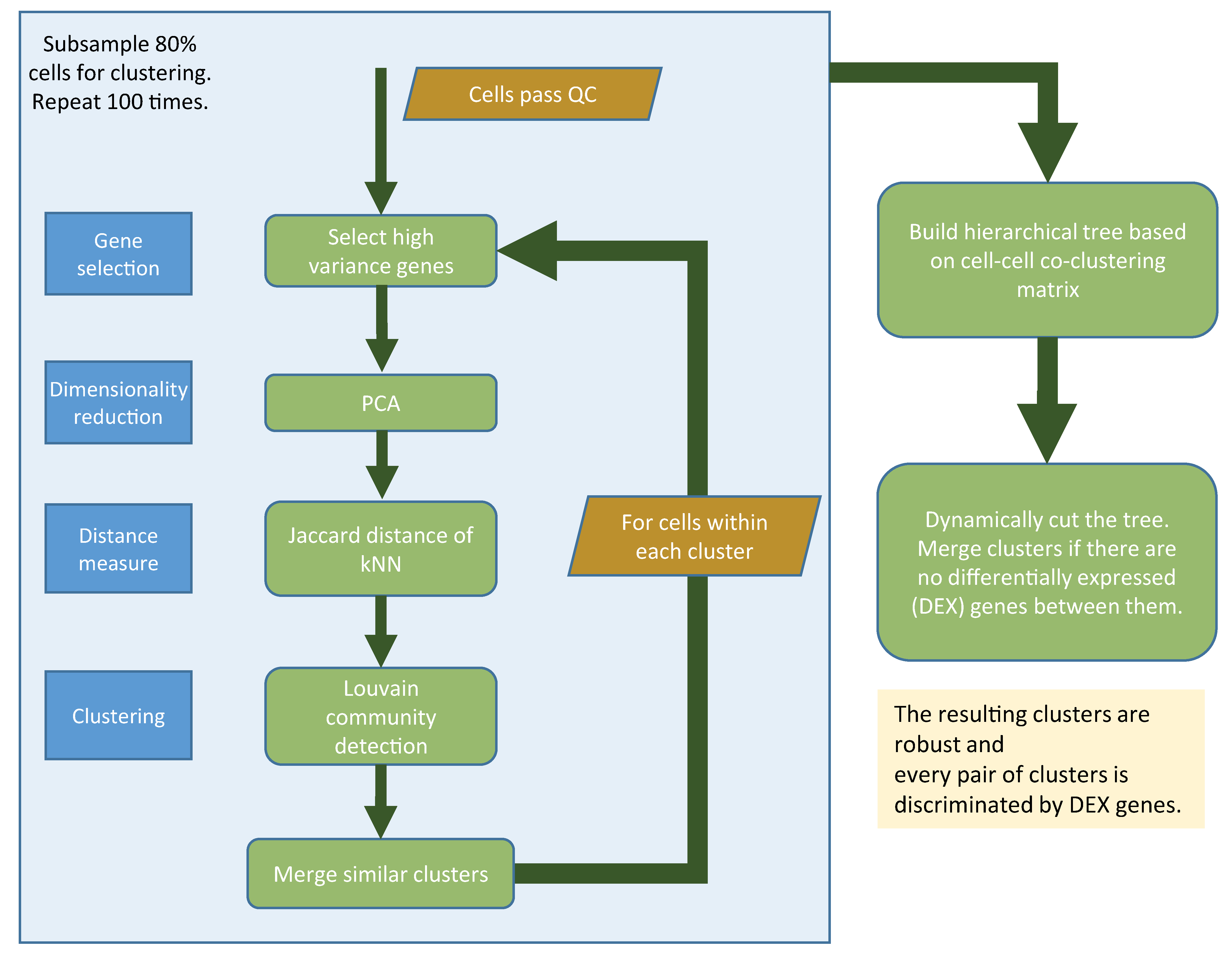

Supplement: S3 Fig — See methods for a detailed description of clustering steps. (TIFF) [file pone.0209648.s003.tiff]

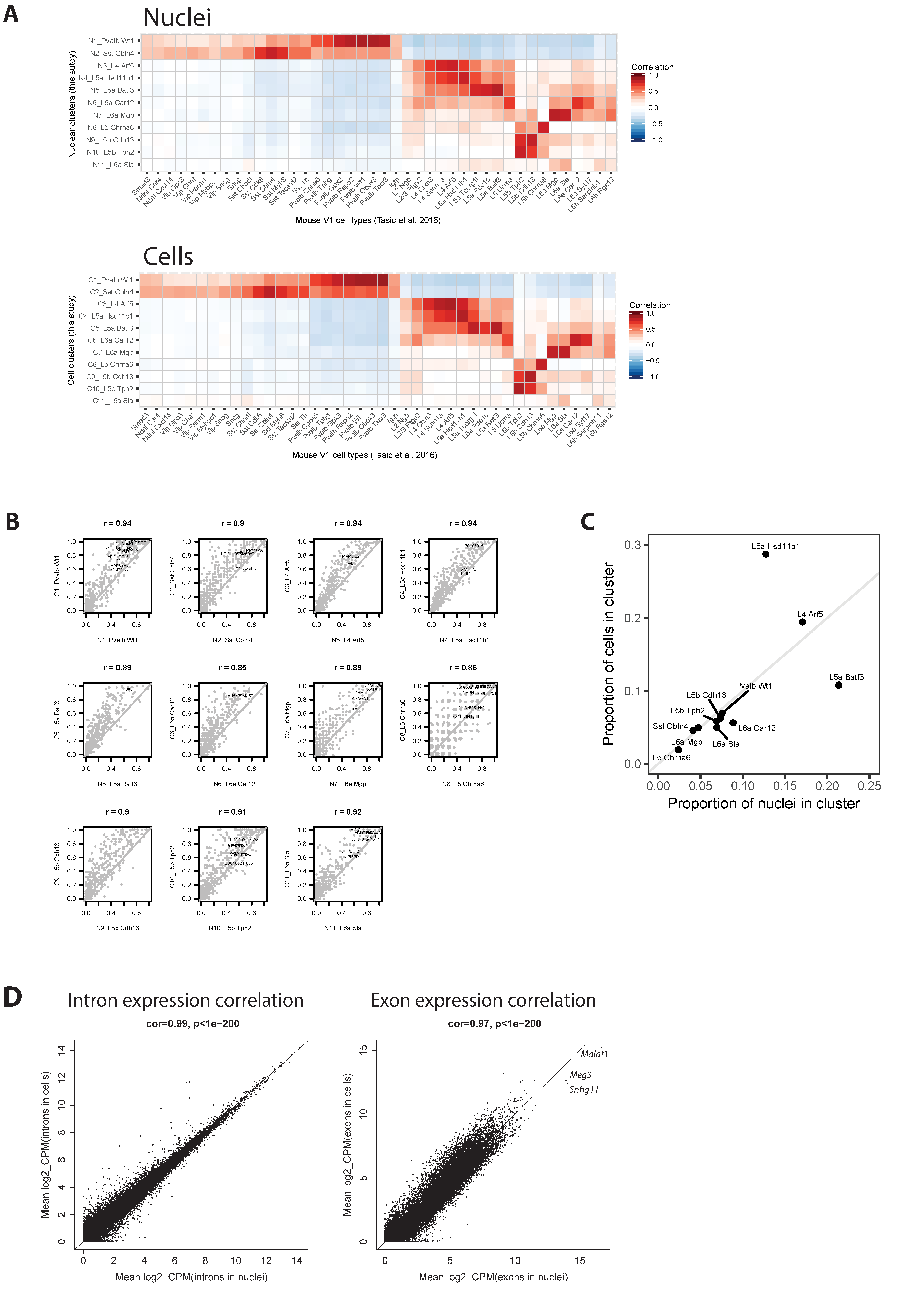

Supplement: S4 Fig — (A) Pairwise correlations between previously reported mouse VISp cell type clusters and nuclear and cell clusters using average cluster expression of the top shared marker genes. Heatmaps show remarkably similar correlation patterns, supporting the existence of a well matched set of nuclear and cell clusters. Nuclear and cell clusters were annotated based on the reciprocal best matching published cluster name and mapped to two interneuron types and five of eight layer 5 excitatory neuron types. (B) Comparisons of the proportion of nuclei or cells expressing marker genes (CPM > 1) for matched pairs of clusters. Correlations are reported at the top of each scatter plot, and cell type specific markers are labeled. As expected based on Fig 2C, gene detection is consistently higher in cells than nuclei. (C) Matched clusters have similar proportions of nuclei and cells (except for two closely related cell types, L5a Hsd11b1 and L5 Batf3), which supports the accuracy of the initial correlation based mapping of single nuclei to cells. (D) Average gene expression quantified based on intronic reads is more highly correlated between cells and nuclei than expression quantified based on exonic reads, particularly for highly expressed genes. Malat1, Meg3, and Snhg11 are the three highest expressing genes in nuclei and have consistently lower expression in cells, as expected based on their reported nuclear localization. (TIFF) [file pone.0209648.s004.tiff]

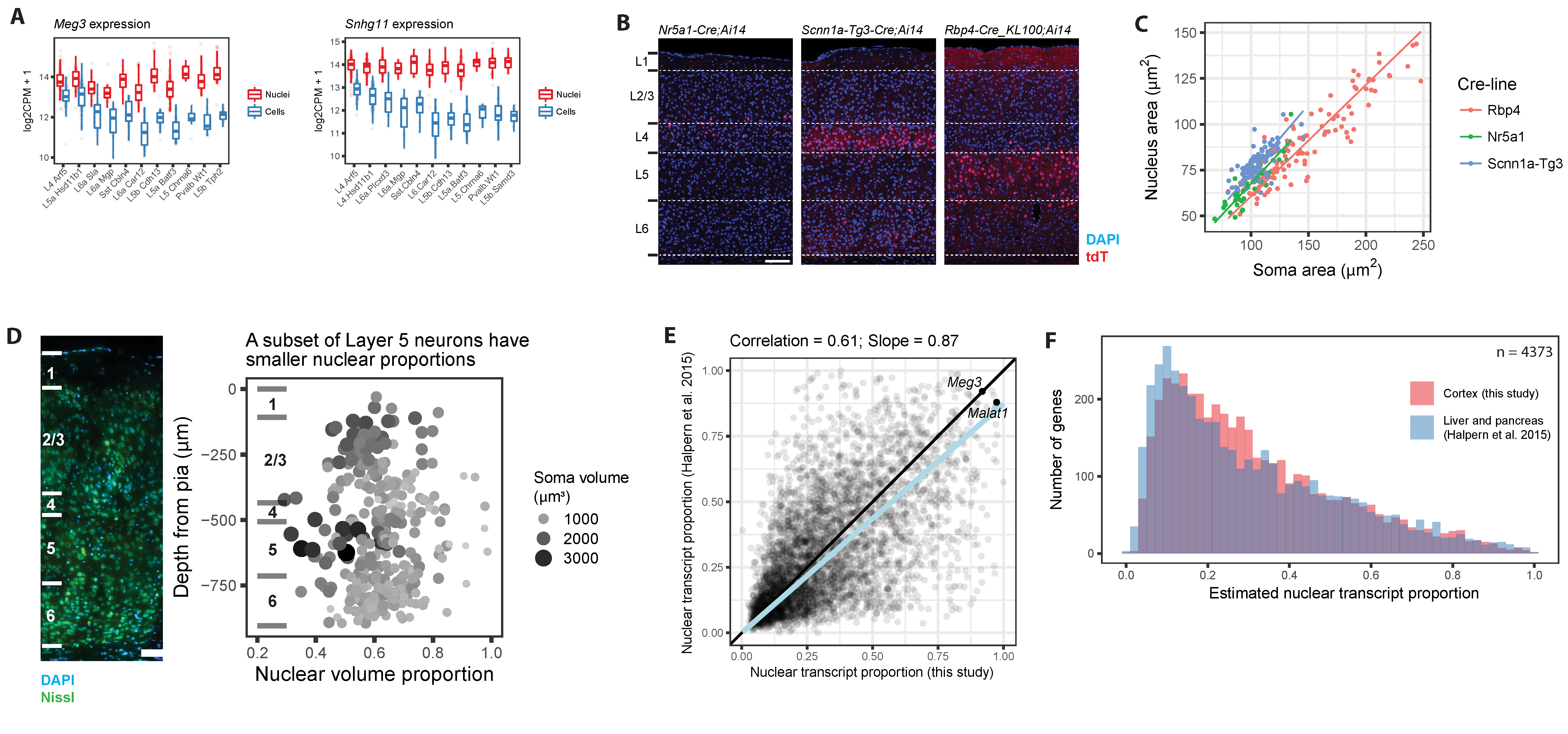

Supplement: S5 Fig — (A) Box plots of log2-transformed expression of two nuclear transcripts, Meg3 and the small nucleolar RNA Snhg11, in matched nuclear and cell clusters. (B) Representative sections of VISp from three Cre-driver mouse lines with layer boundaries, nuclei labeled with DAPI (blue), and subsets of neurons labeled with tdTomato (red). Scale bar is 100 μm. (C) Nucleus and soma area measurements from three Cre-lines, and linear regressions to estimate nuclear proportions. (D) Left: Section of VISp from wild type mouse labeled with DAPI and Neurotrace 500 fluorescent Nissl stain with layer boundaries indicated by white lines. Scale bar is 100 μm. Right: Nuclear volume proportion was quantified based on nucleus and soma area measurements and plotted as a function of cortical depth. Size and color of points are proportional to soma volume. (E) Average nuclear proportions of 4,373 genes (mostly house-keeping) also expressed in mouse pancreatic beta-cells and liver cells are moderately correlated with and approximately 13% less than estimated proportions in this study. (F) The distributions of nuclear proportions are highly similar with slightly higher reported cytoplasmic enrichment for reported genes. Note that the matched set of genes includes 99% protein-coding genes so the distributions more closely resemble those genes in Fig 5D. (TIFF) [file pone.0209648.s005.tiff]
